# Exploring the Wound Healing Potential of Hispidin

**DOI:** 10.3390/nu16183161

**Published:** 2024-09-19

**Authors:** Yi-Shan Liu, Mei-Chou Lai, Tang-Yao Hong, I-Min Liu

**Affiliations:** 1 Department of Dermatology, E-Da Hospital, I-Shou University, Kaohsiung City 84001, Taiwan; 2 School of Chinese Medicine for Post Baccalaureate, College of Medicine, I-Shou University, Kaohsiung City 84001, Taiwan; 3 Department of Pharmacy and Master Program, College of Pharmacy and Health Care, Tajen University, Yanpu Township, Pingtung County 90741, Taiwan; meei@tajen.edu.tw; 4 Department of Environmental Science and Occupational Safety and Hygiene, Graduate School of Environmental Management, College of Pharmacy and Health Care, Tajen University, Yanpu Township, Pingtung County 90741, Taiwan; tyhong@tajen.edu.tw

**Keywords:** hispidin, medicinal mushrooms, wound healing, safety

## Abstract

Background: Hispidin, a polyphenol component mainly derived from the medicinal mushroom species *Phellinus* and *Inonotus*, shows promise for biomedical applications, yet its potential in wound healing remains largely unexplored. This research investigates the wound healing effects of hispidin through in vitro and in vivo experiments, while also evaluating its antimicrobial properties and safety profile. Methods: In vitro scratch assays were conducted to evaluate the impact of hispidin on the migration of NIH-3T3 cells. The wound healing potential of hispidin was assessed in rats using excision wounds, dead space wounds, and linear incisions, treated with various topical ointments including a simple ointment, 2.5% (*w*/*w*) and a 5% (*w*/*w*) hispidin ointment, and a 0.2% (*w*/*w*) nitrofurazone ointment, administered at 0.2 g daily for 14 days. Results: Hispidin demonstrated antimicrobial properties and was particularly effective against *Staphylococcus epidermidis*. Hispidin enhanced NIH-3T3 cell viability, and promoted wound closure in scratch assays, correlating with increased levels of FGF21, TGF-β1, EGF, and VEGF. In excision wound models, the 5% (*w*/*w*) hispidin ointment improved wound contraction, epithelialization, tissue regeneration, fibroblast activity, and angiogenesis. In the granulation tissue from dead space wound models, hispidin reduced pro-inflammatory cytokines (TNF-α, IL-6, IL-1β) and lipid peroxidation, while increasing anti-inflammatory cytokines (IL-10) and antioxidant activities (SOD, GPx, CAT), along with connective tissue markers like hydroxyproline, hexosamine, and hexuronic acid. Hispidin also enhanced wound breaking strength in incision models. Acute dermal toxicity studies indicated no adverse effects at 2000 mg/kg. Conclusions: These findings highlight hispidin’s potential in wound care, demonstrating its antimicrobial, regenerative, and safety properties.

## 1. Introduction

The skin is the body’s largest organ, serving as a protective barrier [1]. Wounds disrupt the skin’s integrity, and the healing process involves four stages: hemostasis, inflammation, proliferation, and remodeling [2]. Each stage is essential for restoring tissue function and structure. Hemostasis initiates healing through vasoconstriction, platelet aggregation, and collagen adhesion [2]. The inflammatory phase involves removing damaged cells and pathogens, with neutrophils and monocytes releasing enzymes and growth factors [3]. In the proliferation phase, reepithelialization, neovascularization, and connective tissue form through fibroblast activity [4]. The remodeling phase enhances collagen fiber organization and increases tissue strength [2].

Open wounds are at risk of bacterial infections, which can lead to systemic infections [5]. Infected wounds heal more slowly and may produce unpleasant fluids and toxins, hindering the regeneration of cells [5]. While many wound treatments focus on preventing inflammation and microbial growth, they often do not promote healing [6]. Antibiotics have been the primary treatment for wound infections, but resistance and adverse reactions require alternative therapies [7]. Researchers are exploring natural compounds and traditional medicines that reduce microbial load and promote healing through their anti-inflammatory and antioxidant properties [8].

Mushrooms have long been valued in folk and traditional medicines for their unique medicinal properties. Extracts from species in the Phellinus and Inonotus genera have been used to treat a wide range of ailments, including malignant tumors, cardiovascular and liver diseases, diabetes, and more [9,10]. These mushrooms are rich in bioactive compounds, such as polysaccharides, triterpenoids, and polyphenols, which contribute to their therapeutic effects [9,10]. Hispidin, a polyphenolic substance, identified as 6-(3,4-dihydroxystyryl)-4-hydroxy-2-pyranone, is a yellowish pigment isolated from the medicinal mushrooms *Inonotus* obliquus and *Phellinus linteus* [11]. According to an earlier study, hispidin has antioxidant activity that is approximately 3–5 times more effective than water-soluble vitamin E, allowing it help protect cells from oxidative stress and reduce damage caused by free radicals [12]. Additionally, several other studies have shown that hispidin possesses anticancer, antibacterial, antihyperglycemic, and neuroprotective properties [11]. Hispidin has been found to have anti-inflammatory properties through its ability to reduce the activation of nuclear factor kappa B, a key player in inflammatory responses, which may help decrease inflammation caused by macrophages [13]. In addition to its medicinal benefits, hispidin is utilized in cosmetics due to its ability to effectively penetrate the skin through advanced drug delivery systems [14]. Furthermore, numerous assessments, including the Ames test, in vitro chromosome aberration test, acute oral toxicity test, and bone marrow micronucleus test, have demonstrated that hispidin exhibits low toxicity and is safe for human use [15].

While hispidin shows great potential as an ingredient for skincare products, owing to its distinctive antioxidant and anti-inflammatory properties [13,14], its specific effects on skin wounds have not been thoroughly researched. Further investigation is needed to fully understand its potential applications in wound healing and skin repair, which could lead to innovative dermatological treatments. Therefore, this research aims to explore the wound-healing properties of hispidin through a combination of in vitro and in vivo experiments.

The timeline for this research includes three main phases. The first phase, from week 1 to week 7, focused on experiment design, including defining research objectives, conducting a literature review, developing the experimental design, and obtaining ethics approval if required. The next phase, from week 8 to week 12, involved preliminary hypothesis testing and analysis. The final phase, from week 13 to week 24, involved executing the full-scale experiment, collecting and analyzing data, and making necessary revisions to the hypothesis or experimental design.

## 2. Materials and Methods

### 2.1. Antimicrobial Activity

The agar well diffusion method was utilized to assess antimicrobial activity [16]. The test microorganisms used to assess antimicrobial activity were *Staphylococcus aureus* (ATCC 29213), *Staphylococcus epidermidis* (ATCC 12228), *Escherichia coli* (ATCC 23815), *Candida albicans* (ATCC 90028), *Candida tropicalis* (ATCC 20401), and *Aspergillus niger* (ATCC 1015). These organisms were procured from the Bioresource Collection and Research Center at the Food Industry Research and Development Institute (Hsinchu, Taiwan). Bacteria were cultured overnight at 37 °C in Mueller Hinton Broth (Sigma–Aldrich, Saint Louis, MO, USA, #90922) and fungi at 28 °C for 72 h in Potato Dextrose Broth (Sigma–Aldrich, Saint Louis, MO, USA, #P6685) and used as inoculum. A final inoculum used 100 µL of suspension containing 10^8^ CFU/mL of bacteria and 10^4^ spore/mL of fungi spread on Mueller Hinton Agar (Sigma–Aldrich, Saint Louis, MO, USA, #103872) and Potato Dextrose Agar (Sigma–Aldrich, Saint Louis, MO, USA, #70139) medium, respectively. The disc (6 mm in diameter) was impregnated with 10 μL of 100 mg/mL (1 mg/disc) hispidin (Santa Cruz Biotechnology, Inc., Santa Cruz, CA, USA, #sc-203998; purity: 98%) placed on seeded agar. Gentamicin (10 µg/disc, #G1914) and tetracycline (10 µg/disc, #T3258) obtained from Sigma–Aldrich, Inc. (Saint Louis, MO, USA) were used as positive controls for bacteria and fluconazole (10 µg/disc, #F8929) and ketoconazole (10 µg/disc, #K1003) obtained from Sigma–Aldrich, Inc. (Saint Louis, MO, USA) for fungi. The test plates were incubated at 37 °C for 24 h for bacteria and at 28 °C for 72 h for fungi, depending on the incubation time required for visible growth. The minimum inhibitory concentration (MIC) values were determined for microorganisms that exhibited sensitivity to hispidin in the disc diffusion assay. 

### 2.2. Cell Culture

NIH/3T3 fibroblast cells, sourced from the American Type Culture Collection, were maintained in Dulbecco’s Modified Eagle Medium (DMEM) and enriched with 10% fetal bovine serum (FBS), 100 U/mL penicillin, and 100 mg/mL streptomycin. The cell cultures were incubated at 37 °C in a humidified atmosphere containing 5% CO_2_ and 95% air. Thw passaging of cells occurred every 3 days, and they were utilized for experiments once they reached approximately 85% confluency.

### 2.3. In Vitro Cell Viability Assay

Cell viability was determined based on the Cell Counting Kit-8 (CCK-8) test (Cat. #96992) following the manufacturer’s protocol (Sigma–Aldrich, Saint Louis, MO, USA). The experiment involved inoculating cells into 96-well plates at a density of 2 × 10^4^ cells/mL. The cells were exposed to different concentrations of hispidin (5, 10, 20, and 40 µmol/L) in the maintenance medium for a period of 24 h. This was done in line with previous studies which have demonstrated that hispidin can help to mitigate the loss of cell viability caused by 1-methyl-4-phenylpyridinium [17]. After treatment, 10 μL of the CCK-8 reagent was added to each well, and the optical density (OD) of each well at 450 nm was measured with a multifunction microplate reader (SpectraMax M5, Molecular Devices, Sunnyvale, CA, USA) after incubation for 2 h at 37 °C. Hispidin was solubilized in dimethyl sulfoxide (DMSO, Sigma–Aldrich, Saint Louis, MO, USA; Cat. #D8418) to create a stock solution, which was subsequently diluted in fresh medium at the appropriate concentrations immediately before use. The control group received only the vehicle, ensuring that the final concentration of DMSO in the culture media remained below 0.1% (*v*/*v*), which did not induce severe cytotoxicity [18]. The viability of the treatment group was represented as a percentage of the control group that received the vehicle treatment.

### 2.4. In Vitro Scratch Assay

The in vitro scratch wound healing assay was employed to evaluate cell migration [19]. NIH/3T3 cells were seeded in 6-well plates at a density of 2 × 10^5^ cells/mL and grown in complete medium until they reached 80–90% confluence. After achieving the desired confluence, the cells were incubated for 24 h at 37 °C with 5% CO_2_. A consistent scratch was made across the cell monolayer using a sterile 200 μL pipette tip, followed by washing away any cellular debris with phosphate-buffered saline. The cells were then treated with various concentrations of hispidin (5, 10, 20, and 40 µmol/L) and allowed to culture for either 12 or 24 h. The extent of scratch closure was observed under an inverted microscope (Olympus, Tokyo, Japan) and quantified using ImageJ 1.38 software (NIH, Bethesda, MD, USA).

### 2.5. Estimation of Growth Factors

After 12 h of conducting the scratch assay, the culture supernatant from all wells was combined and analyzed for the levels of fibroblast growth factor (FGF)21 (#ab223589), transforming growth factor (TGF)-β1 (#ab119557), epidermal growth factor (EGF) (#ab234560), and vascular endothelial growth factor (VEGF) (#ab209882) using commercially available enzyme-linked immunosorbent assay (ELISA) kits, according to the manufacturer’s instructions (Abcam Inc., Cambridge, MA, USA). The absorbance at 450 nm for each well was determined using a multifunction microplate reader (SpectraMax M5, Molecular Devices, Sunnyvale, CA, USA).

### 2.6. Experimental Animals

Male and female Wistar rats, weighing between 200 ± 8.9 g and 250 ± 10.3 g and aged 6–8 weeks, were obtained from the National Laboratory Animal Center in Taipei City, Taiwan. The animals were housed in an environment with a regulated temperature of 25 ± 1 °C and maintained on a 12-h light–dark cycle, with lights turning on at 06:00 h. In our animal center, they had continuous access to food and water. The rats underwent a 7-day acclimatization period in the laboratory setting prior to the commencement of any experimental procedures. All animal procedures adhered strictly to the guidelines set forth by the Animal Welfare Act and the Guide for the Care and Use of Laboratory Animals of the National Institutes of Health. The studies were approved by the Institutional Animal Care and Use Committee (IACUC) at Tajen University (approval number: IACUC 113-9; approval date: 18 January 2024).

The sample size for this study was determined based on both theoretical considerations and practical constraints. In the in vivo wound healing experiments, a total of 96 rats were used across three different models (excision, dead space, and incision), with each model involving 32 rats divided into four groups (simple ointment, 0.2% nitrofurazone ointment, 2.5% hispidin ointment, and 5% hispidin ointment). Additionally, 20 rats were randomly assigned to two groups for the limit test, bringing the total number of rats used in this study to 116. Only healthy animals were included, and any that did not adhere to the experimental protocol or exhibited inconsistent behavior were excluded. To ensure fairness in group assignments and to reduce bias, animals were randomly assigned to experimental groups. Various strategies were also employed to minimize potential confounders, such as randomizing treatment order, maintaining consistent measurement times, and rotating cage locations. Blinding was implemented to further minimize bias and enhance the validity of the results.

### 2.7. Ointment Formulation

Simple ointment was prepared according to the British Pharmacopoeia formula using 170 g of white soft paraffin, 10 g of hard paraffin, 10 g of cetostearyl alcohol, and 10 g of wool fat [20]. To formulate treatment ointments containing 2.5% *w*/*w* and 5% *w*/*w* hispidin, 2.5 g and 5 g of hispidin were incorporated into 97.5 g and 95 g of simple ointment, respectively. Nitrofurazone ointment (0.2% *w*/*w*; GSK Pharmaceuticals, Bangalore, India) was used as a reference drug to assess the wound-healing capabilities of the hispidin ointment.

### 2.8. Acute Dermal Toxicity Test

Healthy adult Wistar rats weighing between 200 and 280 g, and of both sexes, were housed individually and allowed to acclimate to laboratory conditions for 5 days prior to the commencement of acute toxicity studies aimed at determining a safe dosage in accordance with OECD guidelines [21]. Twenty-four hours before the study began, 10% of the dorsal trunk surfaces of the test animals were shaved under appropriate anesthesia. In a limit test, 20 rats were randomly assigned to two groups, each consisting of five males and five females, and given a single dose of 2000 mg/kg following the OECD Guideline 402 (Acute Dermal Toxicity: Fixed Dose Procedure) [21]. The control group received a topical application of a simple ointment, while the test group was treated with an ointment containing 5% *w*/*w* hispidin. Both treatments were administered once at a dosage of 2000 mg/kg. Within an hour of applying the ointment, the rats were monitored for toxic skin reactions including irritation, itching, redness, swelling, and other behavioral changes. These symptoms were documented, and the rats were further observed daily for any adverse skin reactions over a period of 14 days.

### 2.9. In Vivo Wound Healing Activity

To assess the wound healing efficacy of hispidin, three distinct wound models were employed: excision wounds, dead space wounds, and paravertebral linear incisions. The experimental animals in each study were divided into four groups, with each group consisting of eight animals: Group I—treated with a simple ointment; Group II—serving as the positive control, treated with 0.2% *w*/*w* nitrofurazone (NF) ointment; Group III—treated with an ointment containing 2.5% *w*/*w* hispidin; and Group IV—treated with an ointment containing 5.0% *w*/*w* hispidin.

#### 2.9.1. Excision Wound Model

Four groups with eight animals in each group were anesthetized by the open mask method with anesthetic ether. The rats were depilated on the back. A single excision wound, designated as day “0”, was created by cutting away a 500-mm^2^ full thickness of skin from a predetermined area. The wound was left uncovered in an open environment. Subsequently, various ointments were applied topically to wounds created on the dorsal back of rats each day until complete healing occurred. These ointments included simple ointment, a standard ointment (0.2% *w*/*w* nitrofurazone ointment), hispidin ointments at concentrations of 2.5% and 5% (*w*/*w*), and with an approximate dose of 0.2 g per wound. The wound size was assessed by outlining it on millimeter-scale graph paper every second day. Wound contraction was determined as the percentage decrease in wound area over time [22]. The duration of epithelialization was assessed by counting the days needed for the eschar to detach completely, leaving no exposed wound [23].

#### 2.9.2. Dead Space Wound Model

The rats were anesthetized, and a 1 cm incision was created on the dorsolumbar region of their backs. Two polypropylene tubes, each measuring 0.5 × 2.5 cm^2^, were inserted into the dead space in the lumbar region of the rat on both sides, and the wounds were then closed using suture material [24]. The wounds were treated topically with simple ointment, standard nitrofurazone (0.2% *w*/*w*) ointment, and hispidin ointments at concentrations of 2.5% (*w*/*w*) and 5% (*w*/*w*), with each wound receiving a daily application of 0.2 g of the respective ointment. The animals were euthanized on the 3rd, 7th, and 10th days following the injury. The granulation tissue that developed on and around the implanted tubes was meticulously removed and analyzed to assess the levels of pro-inflammatory and anti-inflammatory cytokines, oxidative stress-related malondialdehyde (MDA), antioxidant activity, and collagen tissue characteristics.

#### 2.9.3. Incision Wound Model

Two parallel 6 cm paravertebral incisions were made through the full thickness of the skin, 1 cm lateral to the midline of vertebral column after giving anesthesia [4]. The parted skin was sutured with Acos™ disposable stainless steel skin staplers (Sunmedix Co., Ltd., Namyangju-si, Republic of Korea) at 1 cm intervals. The wounds were treated by topically applying simple ointment, nitrofurazone (0.2% *w*/*w*) ointment, and hispidin ointments at concentrations of 2.5% (*w*/*w*) and 5% (*w*/*w*). Each wound received a daily dose of 0.2 g of ointment for a duration of 14 days. The skin staplers were removed on 10th post-wounding day with continued ointment application. On the 14th day, the animals were euthanized under anesthesia, and the tensile strength of the skin wounds was measured using a tensiometer, determining the weight in grams needed to break an area (mm^2^) of the skin [24]. The force required to cause rupture in the area of an incised wound, measured in grams per square millimeter (g/mm^2^), was defined as the wound breaking strength (WBS).

### 2.10. Estimation of Inflammatory Markers and Antioxidant Activity

On the third day following the creation of the dead space wound model, granulation tissue was harvested from the implanted tubes in each group. This was performed to assess the concentrations of pro-inflammatory and anti-inflammatory cytokines. Seven days after creating the dead space wound model, granulation tissue that formed around the implanted tubes was utilized to evaluate MDA levels and antioxidant activity. Granulation homogenate was prepared using 0.15 mol/L KCl and centrifuged at 8000 rpm for 10 min. The resulting cell-free supernatant was obtained to perform the following studies.

Commercial Enzyme-linked immunosorbent assay (ELISA) kits for tumor necrosis factor (TNF)-α (#ab236712), interleukin (IL)-6 (#ab234570), IL-1 β (#ab255730), and IL-10 (#ab214566) were obtained from Abcam Inc. (Cambridge, MA, USA) to measure cytokine levels expressed as pg/mg protein. The assays were performed according to the manufacturer’s instructions, and the absorbance at 450 nm was determined using a microplate reader (SpectraMax M5, Molecular Devices, Sunnyvale, CA, USA).

The levels of MDA were determined using a competitive ELISA kit (#ab238537) as per the manufacturer’s instructions, provided by Abcam Inc. of Cambridge, MA, USA. The concentration of MDA was calculated by measuring the absorbance at 450 nm using a microplate reader (SpectraMax M5, Molecular Devices, Sunnyvale, CA, USA). The results were expressed in nmol/mg protein.

The antioxidant activity assay was determined by the colorimetric method. Colorimetric assay kits for superoxide dismutase (SOD; #ab65354), glutathione peroxidase (GPx; #ab102530), and catalase (CAT; #ab83464) were obtained from Abcam Inc. (Cambridge, MA, USA). The activities of SOD, GPx, and CAT were measured by recording absorbance at 450 nm, 340 nm, and 570 nm, respectively, using a SpectraMax M5 microplate reader (Molecular Devices, Sunnyvale, CA, USA). Enzymatic activities were standardized and reported in units per milligram of protein.

To determine the total protein in the sample, the BCA protein assay kit (#ab102536) from Abcam Inc. (Cambridge, MA, USA). The protein concentration was assessed by monitoring the color change of the sample solution, which shifts from green to purple. This color transition correlates with protein concentration and occurs within the absorbance spectrum centered at 562 nm. Each experiment was conducted in triplicate to validate the results.

### 2.11. Estimation of Connective Tissue Parameters

At day 10 post-wounding in the dead space wound model, granulation tissue formed on the implanted tubes were collected to estimate the connective tissue parameters. To prepare the sample, 40 mg of dried granulation tissue was placed into each tube and 1 mL of 6N HCl was added. The tubes were then hydrolyzed and kept in a boiling water bath for 12 h each day over 2 consecutive days. After hydrolysis, the hydrolysate was cooled, and excess acid was neutralized using 10N NaOH with phenolphthalein as an indicator. Subsequently, the volume of neutral hydrolysate was diluted to a concentration of 20 mg/mL with distilled water. This final hydrolysate was used to estimate the concentration of hydroxyproline, hexosamine, and hexuronic acid using commercially available ELISA assay kits. The hydroxyproline ELISA kit (#ab222941) was obtained from Abcam Inc. (Cambridge, MA, USA) and operates within the absorbance band centered at 562 nm. The hexosamine ELISA kit (#MBS3809347) was obtained from MyBioSource, Inc. (San Diego, CA, USA), while the hyaluronic acid ELISA kit (#A75464) was purchased from Antibodies.com (accessed on 26 February 2024) (Cambridge, UK). The absorbance readings at 450 nm used a microplate reader (SpectraMax M5, Molecular Devices, Sunnyvale, CA, USA) to determine the hexosamine and hyaluronic acid concentrations. The results were expressed in μg/mg protein.

### 2.12. Histopathological Studies

Skin samples of the rats from each treatment group of excision wound model were collected on 14th post-wounding day for further histopathological studies. The skin samples were treated with a 10% neutral-buffered formalin solution, and the solution was replaced every 2 days until the tissues solidified. Each sample was then enclosed in a paraffin block, and thin sections measuring 3 μm were created. These sections were stained with hematoxylin and eosin (H&E) for general morphological observations. The slides were examined using a Axio Imager M2 light microscope (Carl Zeiss Nts, LLC. Peabody, MA, USA) to evaluate various aspects such as re-epithelialization, collagen formation, fibroblast proliferation, angiogenesis, and the formation of granulation tissue. These features were assessed based on their intensity, which was graded 1–4: 1 (minimal 1–25%), 2 (mild 26–50%), 3 (moderate 51–75%), or 4 (high 76–100%) [25].

### 2.13. Statistical Analysis

The data are presented as the mean ± standard deviation (SD). Statistical analysis was conducted using one-way ANOVA with SigmaPlot Version 14.0 (Systat Software Inc., San Jose, CA, USA). Dunnett’s post-hoc comparisons were applied to identify significant differences when applicable. Statistical significance was set at *p* < 0.05.

## 3. Results

### 3.1. Effect on Antimicrobial Activity

Hispidin demonstrated inhibitory effects on the growth of all tested microorganisms, with varying degrees of efficiency observed among different species (Table 1). The MIC of hispidin ranged from 100 to 250 µg/disc. The lowest MIC, 100 µg/disc, was observed for *Staphylococcus epidermidis*, indicating that it had the highest sensitivity to hispidin. For *Candida tropicalis* and *Staphylococcus aureus*, the MIC values were 150 and 200 µg/disc, respectively. Hispidin exhibited an identical MIC value of 250 µg/disc against both *Candida albicans* and *Escherichia coli* (Table 1).

### 3.2. Effect on Cell Viability

**Figure 1:**
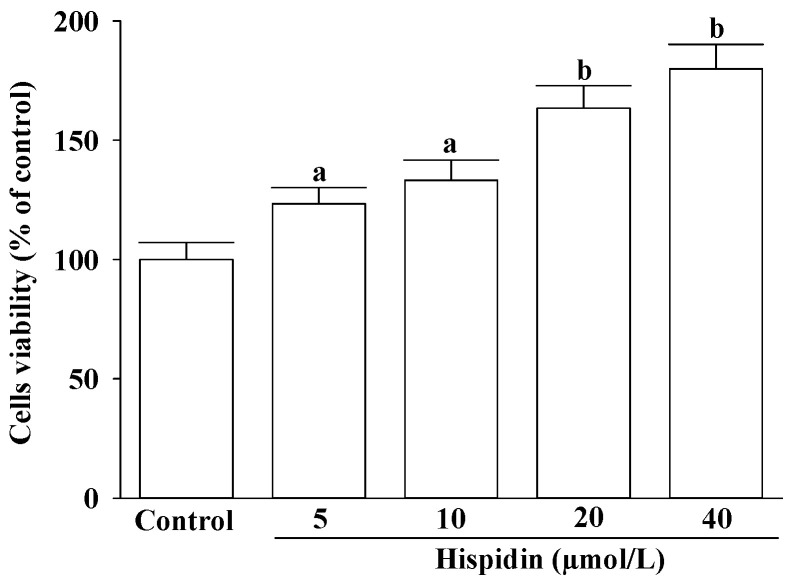
Effect of hispidin on the viability of NIH-3T3 cells. Cells were treated with hispidin at concentrations of 5, 10, 20, and 40 μmol/L for 24 h. Cell viability was assessed using the CCK-8 assay. Data are expressed in mean ± SD of five independent experiments (*n* = 5) performed in triplicate. ^a^ *p* < 0.05 and ^b^ *p* < 0.01 compared to the control group that received the vehicle treatment.

Hispidin enhanced cell viability in a concentration-dependent manner within the range of 5 to 40 μmol/L (Figure 1). At a concentration of 40 μmol/L, hispidin achieved cell viability levels exceeding 80% compared to the control group. This effect was observed over a continuous 24-h testing period, indicating its potential for sustaining cellular health under these conditions (Figure 1).

### 3.3. Effects on the Scratch Wound Closure In Vitro

**Figure 2:**
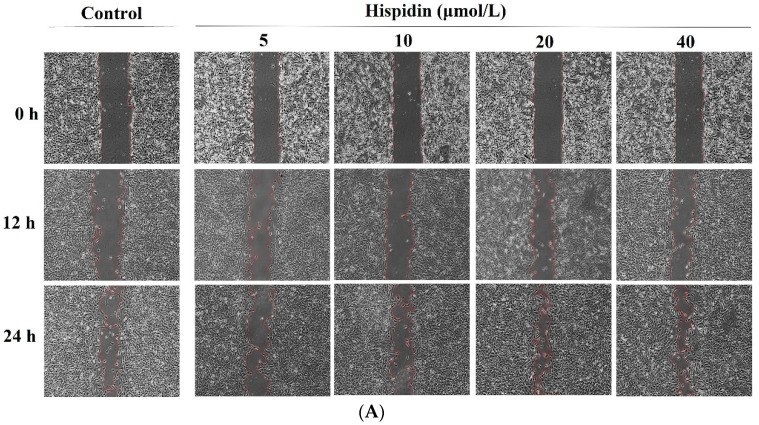

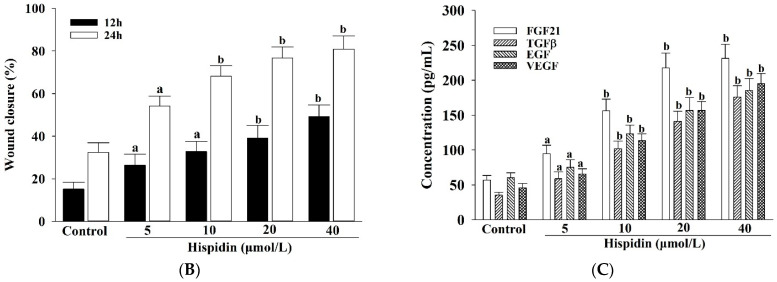
Effect of hispidin on the scratch closure in vitro. (**A**) The cells exhibited migration towards the wound gap after 12 and 24 h of incubation, as illustrated in the representative optical images taken at a magnification of ×100. (**B**) The extent of scratch closure was measured using the ImageJ 1.38 software. (**C**) The levels of FGF21, TGF-β1, EGF, and VEGF in the culture supernatant were assessed 12 h post-scratch assay. The data were expressed as the mean ± SD of five independent experiments (*n* = 5) performed in triplicate. ^a^ *p* < 0.05 and ^b^ *p* < 0.01 compared to the control group that received the vehicle treatment.

As shown in Figure 2A, noticeable cell migration into the scratched area was observed at both 12 and 24 h. In the presence of hispidin, there was a concentration-dependent reduction in the wound area compared to the vehicle control group. After 24 h, a remarkable closure of the scratch facilitated by hispidin was evident. Particularly noteworthy was the effect of 40 μmol/L hispidin on in vitro wound healing, resulting in a wound closure rate of almost 80.9% (Figure 2B).

After 12 h of conducting the scratch assay, it was observed that FGF21 levels in the culture supernatant increased in response to varying concentrations of hispidin. The highest concentration of FGF21 was found when cells were treated with 40 μmol/L hispidin (Figure 2C). Treatment with hispidin led to a concentration-dependent increase in the levels of TGF-β1 and EGF, with a notable elevation observed in the group treated with 40 μmol/L of hispidin (Figure 2C). An increase in hispidin concentration was noted to correspond with elevated VEGF levels across all cell lines, with a particularly pronounced effect observed in the group treated with 40 μmol/L of hispidin (Figure 2C).

### 3.4. Effects on Wound Healing in the Excision Wound Model

#### 3.4.1. Wound Contraction

Table 2 shows the rate of wound contraction in the excision wound model. According to the results, the groups treated with 5% (*w*/*w*) hispidin ointment showed improvement compared to the simple ointment groups and nitrofurazone ointment-treated group from the 3rd day after wounding. On the 14th day post-wounding, both 2.5% (*w*/*w*) and 5% (*w*/*w*) hispidin ointment treatment groups showed similar results, with 92.5 ± 4.7% and 94.6 ± 5.3%, respectively, compared to the simple ointment groups. While both groups treated with 2.5% (*w*/*w*) and 5% (*w*/*w*) hispidin ointment exhibited increased rates of wound contraction, these were not different from the group treated with nitrofurazone ointment (88.9 ± 5.1%) by the 14th day post-wounding.

#### 3.4.2. Period of Epithelialization

The application of hispidin ointment at concentrations of 2.5% (*w*/*w*) or 5% (*w*/*w*) led to a notable reduction in the time required for epithelialization compared to the group treated with a simple ointment (Table 3). There was no difference in the time needed for epithelialization between the groups treated with hispidin and nitrofurazone ointments. Based on the percentage reduction in the epithelialization period (Table 3), it appears that higher concentrations of hispidin may be effective in promoting faster wound healing compared to nitrofurazone.

#### 3.4.3. Histopathological Analysis

**Figure 3:**
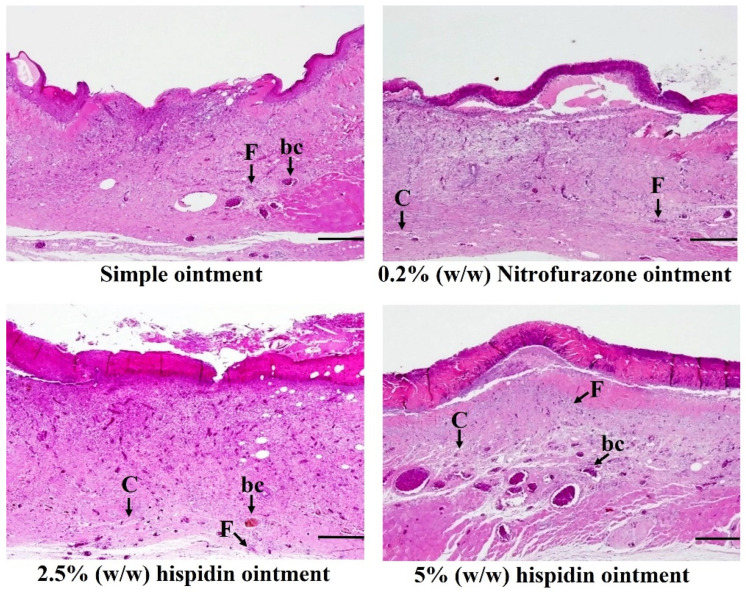
Histological analysis was performed on sections of healed wounds, which were stained with hematoxylin and eosin. Photomicrographs taken on the 14th day post-surgery illustrate the healing progress in rats with excision wounds treated with ointment. The scale bar indicates 100 µm. Abbreviations: bc for blood capillaries, C for collagen fibers, and F for fibroblasts.

The Figure 3 shows the histopathological characteristics of the healed wounds on day 14 after wounding. The wounds in the group treated with simple ointment showed disorganized fibroblasts, reduced collagen fiber deposition, and limited angiogenesis. In contrast, the wounds treated with either nitrofurazone or hispidin ointment exhibited less scar formation, enhanced fibroblast proliferation, and the presence of newly formed blood capillaries. Moreover, the upper thick layer of cells observed in the group treated with hispidin and nitrofurazone ointment shows greater re-epithelialization. The characteristic histopathological features of the healed wounds in the experimental animals, along with their respective scores, are presented in Table 4, which indicates that there was no difference in effect between nitrofurazone and the 5% (*w*/*w*) or 2.5% (*w*/*w*) hispidin ointment based on the histopathological analysis scores.

### 3.5. Effects on Dead Space Wound Model

#### 3.5.1. Effects on Inflammatory and Anti-Inflammatory Cytokines in Granulation Tissues

In the dead space wound model, 3 days after wound formation, rats treated with simple ointment exhibited higher levels of TNF-α, IL-6, and IL-1β in their granulation tissue compared to those treated with hispidin or nitrofurazone ointments. In rats treated with hispidin ointment, those receiving a 5% (*w*/*w*) concentration showed a notable reduction in TNF-α, IL-6, and IL-1β levels compared to those treated with a 2.5% (*w*/*w*) concentration (Table 5). Additionally, 3 days after inducing wounds in the dead space wound model, rats treated with either hispidin or nitrofurazone ointments showed elevated IL-10 levels in the granulation tissue compared to those treated with a plain ointment (Table 5). The efficacy of hispidin ointment in elevating IL-10 levels was particularly evident at a 5% (*w*/*w*) concentration (Table 5).

#### 3.5.2. Effects on Lipid Peroxidation and Antioxidant Activity in Granulation Tissues

Seven days after wound formation in the dead space wound model, elevated levels of MDA were observed in the granulation tissue (Table 5). Nevertheless, the groups treated with hispidin or nitrofurazone ointment exhibited lower MDA levels compared to the control group that received the sample ointment (Table 5). Furthermore, 7 days after the wound in the dead space wound model, there was a noticeable decrease in the activity of SOD and GPx, as well as a reduction in CAT content within the granulation tissue. Nevertheless, this reduction was mitigated in rats treated with hispidin or nitrofurazone ointment compared to those in the control group receiving simple ointment (Table 5). In the group treated with hispidin, the application of a 5% (*w*/*w*) hispidin ointment decreased MDA levels and was more effective in enhancing the activities of SOD and GPx, as well as increasing CAT content, compared to the group using a 2.5% (*w*/*w*) hispidin ointment (Table 5).

#### 3.5.3. Effects on Connective Tissue Parameters in Granulation Tissues

On day 10 post-wounding in the dead space wound model, the groups treated with either hispidin or nitrofurazone ointment showed an increase in connective tissue parameters, including hydroxyproline, hexosamine, and hexuronic acid, compared to the group that received a sample ointment (Table 5). Within the group receiving hispidin treatment, those treated with 5% (*w*/*w*) hispidin ointment exhibited higher levels of these connective tissue parameters compared to those treated with 2.5% (*w*/*w*) hispidin ointment (Table 5).

### 3.6. Effects on Wound Breaking Strength in Incision Wound Model

Rats treated with 0.2% (*w*/*w*) nitrofurazone ointment demonstrated an increase in WBS, measuring 387.6 ± 8.2 g/mm^2^ on the 14th day post-injury, which was higher compared to those treated with a simple ointment (282.3 ± 6.9 g/mm^2^). The WBS in rats treated with 2.5% (*w*/*w*) and 5% (*w*/*w*) ointments were 375.2 ± 9.7 g/mm^2^ and 404.6 ± 10.3 g/mm^2^, respectively. Both values are higher than the WBS observed in rats treated with the sample ointment. The WBS percentage increased by 37.2% for the 0.2% nitrofurazone ointment group, 32.9% for the 2.5% (*w*/*w*) hispidin ointment group, and 43.3% for the 5% (*w*/*w*) hispidin ointment group (Table 6).

### 3.7. In Vivo Acute Dermal Toxicity Studies

In the acute toxicity study, rats were given either the sample ointment or a 5% (*w*/*w*) hispidin ointment at a maximum dose of 2000 mg/kg. There were no observed changes in fur, eyes, or mucous membranes, and no adverse skin reactions such as redness, swelling, irritation, itching, or changes in behavior. Additionally, there were no fatalities recorded during the study period.

## 4. Discussion

The healing process of a wound is delayed by pathogen infections, with the primary issue being that wounds are easily contaminated by bacteria [26]. The main bacteria found at injury sites include the predominantly Gram-positive *S. aureus* and *S. epidermidis*, as well as the Gram-negative *E. coli* [27]. Additionally, Candida species, particularly *C. tropicalis* and *C. albicans*, are yeast fungi that often contribute to wound infections, further delaying the healing process as the most commonly identified pathogens in fungi-positive wounds [28]. Our research revealed that hispidin exhibits inhibitory effects on the growth of all tested microorganisms, underscoring its broad-spectrum antimicrobial activity, with especially notable efficacy against *Staphylococcus epidermidis*. Although the detailed antimicrobial mechanisms of hispidin were not clarified in the study, it may inhibit bacterial cell wall synthesis, weakening the walls and disrupting membrane integrity by altering permeability and making bacteria more susceptible to damage, leading to cell lysis [29]. In fungi such as Candida species, hispidin may bind to ergosterol in the fungal cell membrane, disrupting membrane function and causing cell death [30]. Given these properties, hispidin could play a crucial role in preventing harmful microbes from infecting wounds, thereby potentially accelerating the healing process and reducing the risk of complications associated with wound infections.

Fibroblasts play a crucial role in wound healing by promoting re-epithelialization and facilitating wound closure [31]. Therefore, their proliferation and migration are critical for efficient wound repair. Our study reveals that hispidin enhances cell viability and promotes fibroblast migration. This was evidenced by the wound scratch assay conducted on NIH-3T3 cells, a common in vitro method for studying cell migration and proliferation [19]. These results suggest that hispidin supports fibroblast viability and stimulates their proliferation and migration, which are vital for the repair process.

Disturbance of the cell monolayer leads to the disruption of cell–cell contacts. This triggers aggregation and the release of growth factors or cytokines at the wound site, promoting cell migration and proliferation [31]. Critical growth factors involved in this process include FGF, TGF-β, EGF, and VEGF [32,33,34,35]. FGF plays a vital role in stimulating the growth and specialization of fibroblasts and endothelial cells, thus promoting angiogenesis and supporting the healing process of wounds [32]. TGF-β1, secreted by fibroblasts, functions as a versatile growth factor that recruits inflammatory cells, enhances macrophage-mediated tissue debridement, supports angiogenesis, promotes reepithelialization, and increases collagen deposition during the proliferative phase [33]. EGF stimulates fibroblast migration and proliferation, promotes angiogenesis and epithelialization, and induces fibroblasts to secrete growth factors, all of which expedite wound healing [34]. Additionally, VEGF plays a crucial role in triggering angiogenesis and facilitating wound healing [35]. In an in vitro scratch wound healing model, higher levels of FGF, TGF-β1, EGF, and VEGF were associated with hispidin concentration. The results suggest that hispidin has the potential to boost the release of growth factors, which makes it a promising candidate for improving wound healing. However, it is essential to recognize that in vitro assays may only partially replicate the complex process of wound healing in living organisms. As a result, additional validation using animal model studies will be crucial to achieving a deeper understanding of hispidin’s role in wound healing.

Excision wounds are valuable for assessing wound contraction and epithelialization rates [36]. In this study, such models were used to evaluate the effects of hispidin ointment on wound healing. The findings revealed that the hispidin-treated group exhibited smaller wound sizes and a shorter epithelialization period compared to the control group. Hispidin markedly accelerated wound contraction, likely due to increased fibroblast proliferation, enhanced angiogenesis, faster granulation tissue formation, and denser collagen deposition, as supported by our histopathological observations.

Collagen is the main protein found in the tissue that forms during the healing process of a wound [37]. After an injury, the production of collagen in the wound area increases quickly to help strengthen and support the tissue [37]. Measuring hydroxyproline, a substance produced when collagen breaks down, can show how quickly collagen is being renewed [38]. It was found that hispidin can speed up the healing process and increase the strength of healed incision wounds in a concentration-dependent manner. Furthermore, treatment with hispidin resulted in higher levels of hydroxyproline in the granulation tissue of the wound, indicating increased collagen content and renewal. This contributes to faster healing and improved strength in the treated wounds. Hexosamine and hexuronic acid are essential molecules in the extracellular matrix. They provide the foundation for new matrix synthesis by forming a gel-like substance that hydrates and creates a matrix for collagen fibers, stabilizing them by improving interactions that control their alignment and size [39]. Our study revealed that treatment with hispidin raised hexosamine and hexuronic acid levels. This indicates that the observed increase in tensile strength due to hispidin was not only a result of improved collagen synthesis but also of the proper deposition and alignment of collagen.

The progression from the inflammatory phase to the proliferative phase is a critical aspect of wound healing. The inflammatory phase, which occurs 1 to 3 days post-injury, is essential for controlling bleeding, activating the innate immune response to combat pathogens, and removing dead tissue. This phase typically moves into the proliferative phase, which occurs from 4 to 21 days [40]. Although inflammation starts the healing process, too much or prolonged inflammation can slow down healing and increase the risk of chronic, non-healing wounds [41]. Therefore, it is important to maintain a balance of both pro- and anti-inflammatory proteins at the wound site [42]. One important protein involved in maintaining this balance is IL-10. Renowned for its potent anti-inflammatory properties, IL-10 can regulate other inflammatory proteins, such as TNF-α, IL-1β, and IL-6, as well as various other pro-inflammatory substances [43]. Additionally, IL-10 is vital in fetal wound healing and has been shown to enhance the healing of adult skin wounds [44]. Our research shows that applying hispidin ointment increases IL-10 levels while reducing the levels of TNF-α, IL-1β, and IL-6 in granulation tissue by the 3rd day after the wound. This increase in IL-10 decreases the production of other inflammatory proteins, reducing inflammation and improving wound healing by enhancing the formation of new tissue and organizing collagen fibers [44]. The improved rate of wound closure demonstrates the role of hispidin in decreasing long-lasting inflammation during the inflammatory phase and helping with transition to the proliferative phase in wound healing.

Research indicates that low antioxidant levels and high markers of free radical damage delay wound healing [45]. Reactive oxygen species (ROS) generated in response to skin injuries hinder the healing process by damaging cellular membranes, DNA, proteins, and lipids, while antioxidants aid in wound healing [45]. Boosting the activity of antioxidants like SOD, GPx, and CAT, which neutralize free radicals and eliminate ROS in granulation tissues, is crucial for accelerating wound healing and is an essential strategy for treating chronic wounds [45]. Aligning with previous studies that emphasize the strong antioxidant effects of hispidin [12], our research noted a substantial rise in antioxidant levels and a decrease in MDA levels, an indicator of free radical damage, in wounds treated with hispidin. These findings suggest that hispidin facilitates wound healing by protecting against oxidative damage and reducing inflammation through its strong antioxidant properties, ultimately enhancing the overall healing process.

Although hispidin is generally regarded as safe and has potential as a cosmetic ingredient [15], further evaluation is necessary to determine its safety for use in wound healing treatments. An acute toxicity study on rats, using 5% hispidin ointment at a maximum dose of 2000 mg/kg, showed no adverse effects, indicating a high degree of dermatological safety. The ointments did not cause any systemic toxicity or behavioral changes, and the study reported no fatalities. These results suggest that hispidin ointments are safe even at high doses, highlighting hispidin’s potential for development as a skin medication. However, its limited lipid solubility, which affects absorption, may need enhancement through increased lipid solubility or epidermal delivery systems [14]. Addressing these issues will be crucial for hispidin’s development as a wound-healing agent.

Although this study suggests that hispidin could enhance wound healing, several limitations must be considered. First, selection bias is a concern, as in vitro assays and animal models may not accurately reflect human biological responses. Second, the generalizability of these findings is limited because laboratory conditions differ significantly from real-world environments, affecting the relevance of the results to clinical settings. Lastly, the study primarily focused on short-term effects, highlighting the necessity for long-term research to evaluate the ongoing effectiveness and safety of hispidin in wound healing.

## 5. Conclusions 

This research clearly highlights the immense potential of hispidin, a polyphenol derived from fungi, in promoting wound healing through various mechanistic pathways. These include potent antimicrobial effects, reduction of inflammation and oxidative stress, and the active promotion of wound healing processes such as contraction, epithelialization, and tissue regeneration. Additionally, hispidin enhances fibroblast activity, angiogenesis, and connective tissue strength. Its robust safety profile and broad range of beneficial effects solidify its potential as a therapeutic agent in clinical settings, making it a promising candidate for further development and application in wound healing therapies.

## Figures and Tables

**Table 1:** Antimicrobial activity of the tested compounds assessed via the disc diffusion method.

Microorganisms	Zone of Inhibition in Diameter (mm)					MIC Values (µg/disc)
	Tetracycline (10 µg/disc)	Gentamicin (10 µg/disc)	Fluconazole (30 µg/disc)	Ketoconazole (10 µg/disc)	Hispidin (1 mg/disc)	Hispidin
*Staphylococcus aureus*	31.9 ± 0.7	32.8 ± 0.6	–	–	13.7 ± 0.9	200
*Staphylococcus epidermidis*	34.3 ± 0.5	34.7 ± 0.8	–	–	15.8 ± 0.8	100
*Escherichia coli*	23.4 ± 0.6	38.3 ± 0.7	–	–	10.3 ± 0.7	250
*Candida albicans*	–	–	32.9 ± 0.8	33.7 ± 0.7	11.8 ± 0.8	250
*Candida tropicalis*	–	–	39.7 ± 0.9	37.1 ± 0.6	14.6 ± 0.6	150

Values are mean ± SD of three replicate experiments. –: no data vailable.

**Table 2:** Effect of test ointments on wound contraction in excision wound model.

Post-Wounding (Days)	Wound Contraction (%)			
	Simple Ointment	0.2% (*w*/*w*) NF Ointment	2.5% (*w*/*w*) Hispidin Ointment	5% (*w*/*w*) Hispidin Ointment
1	1.3 ± 0.4 ^c^	4.3 ± 1.7 ^a^	2.4 ± 1.2 ^c^	3.9 ± 1.9 ^a^
3	12.8 ± 3.2	15.3 ± 2.8	17.4 ± 5.6	24.0 ± 4.7 ^a,c^
5	17.8 ± 3.9	20.6 ± 3.6	32.2 ± 4.2 ^a,c^	44.7 ± 4.5 ^a,d^
7	24.3 ± 4.3 ^c^	35.8 ± 4.9 ^a^	56.6 ± 3.8 ^b,c^	66.7 ± 5.2 ^b,d^
9	40.5 ± 3.9 ^c^	52.3 ± 5.7 ^a^	74.6 ± 4.1 ^b,c^	79.7 ± 4.3 ^b,c^
11	60.2 ± 5.5 ^c^	75.3 ± 4.2 ^a^	88.5 ± 3.5 ^b,c^	91.6 ± 4.8 ^b,c^
14	79.4 ± 4.2	88.9 ± 5.1	92.5 ± 4.7 ^a^	94.6 ± 5.3 ^a^

Values (mean ± SD) were obtained from each group of eight animals. ^a^ *p* < 0.05 and ^b^ *p* < 0.01 compared to the values of rats treated with simple ointment on the indicated post-wounding day, respectively. ^c^ *p* < 0.05 and ^d^ *p* < 0.01 compared to the values of rats treated with nitrofurazone (NF) ointment on the indicated post-wounding day, respectively.

**Table 3:** Effect of test ointments on period of epithelialization (PE) in excision wound model.

Treatments	PE (days)	Decrease in PE (%)
Simple ointment	23.4 ± 0.7 ^c^	–
0.2% (*w*/*w*) nitrofurazone ointment	17.2 ± 0.6 ^a^	27.6 ± 5.9
2.5% (*w*/*w*) hispidin ointment	16.1 ± 0.7 ^a^	30.2 ± 4.8
5% (*w*/*w*) hispidin ointment	15.8 ± 0.6 ^b^	31.5 ± 5.1

Values (mean ± SD) were obtained from each group of eight animals. ^a^ *p* < 0.05 and ^b^ *p* < 0.01 compared to the values of rats treated with simple ointment, respectively. ^c^ *p* < 0.05 compared to the values of rats treated with nitrofurazone ointment, respectively. –: no data available.

**Table 4:** The histopathological evaluations of healed wounds treated with experimental ointments in an excision wound model on the 14th day post-injury.

Treatments	Re-Epithelialization	Collagen Formation	Fibroblast Proliferation	Angiogenesis	Granulation Formation
Simple ointment	3	3	3	3	3
0.2% (*w*/*w*) nitrofurazone ointment	4	4	4	4	4
2.5% (*w*/*w*) hispidin ointment	3	4	4	4	4
5% (*w*/*w*) hispidin ointment	4	4	4	4	4

Values were obtained from each group of eight animals. The scoring pattern is as follows: 1 (minimal 1–25%), 2 (mild 26–50%), 3 (moderate 51–75%), or 4 (high 76%–100%).

**Table 5:** Effects of test ointments on pro-inflammatory and anti-inflammatory cytokines, lipid peroxidation, antioxidant activity, and collagen tissue parameters in granulation tissue over specified days in an incision wound model.

Post-Wounding (Days)	Parameters	Simple Ointment	0.2% (*w*/*w*) NF Ointment	2.5% (*w*/*w*) Hispidin Ointment	5% (*w*/*w*) Hispidin Ointment
3	TNFα (pg/mg protein)	278.1 ± 16.9 ^d^	194.5 ± 18.8 ^b^	228.1 ± 17.5 ^a^	213.6 ± 20.4 ^a^
	IL-6 (pg/mg protein)	124.2 ± 14.7 ^c^	80.3 ± 15.6 ^a^	96.2 ± 12.6 ^a^	86.5 ± 13.5 ^a^
	IL-1β (pg/mg protein)	50.7 ± 5.9 ^c^	32.1 ± 6.1 ^a^	38.5 ± 5.4 ^a^	34.6 ± 5.0 ^a^
	IL-10 (pg/mg protein)	411.7 ± 23.2 ^d^	670.2 ± 24.5 ^b^	584.0 ± 25.4 ^b,c^	684.3 ± 23.7 ^b,c^
7	MDA (nmol/mg protein)	48.2 ± 4.2 ^d^	24.9 ± 3.1 ^b^	38.6 ± 4.5 ^a,c^	28.2 ± 3.8 ^b^
	SOD (U/mg protein)	5.1 ± 1.5 ^d^	13.2 ± 2.8 ^b^	10.7 ± 2.1 ^a^	12.7 ± 1.9 ^a^
	GPx (U/mg protein)	74.2 ± 8.3 ^d^	134.3 ± 12.1 ^b^	112.5 ± 10.4 ^a^	124.1 ± 14.3 ^b^
	CAT (U/mg protein)	7.5 ± 1.9 ^c^	15.4 ± 2.2 ^a^	10.6 ± 2.1 ^c^	14.5 ± 2.3 ^a^
10	Hydroxyproline (μg/mg protein)	47.3 ± 7.2 ^d^	87.3 ± 9.3 ^b^	70.1 ± 8.4 ^b^	82.2 ± 8.3 ^b^
	Hexosamine (μg/mg protein)	59.6 ± 8.1 ^d^	109.1 ± 12.1 ^b^	87.6 ± 9.7 ^b^	102.8 ± 10.3 ^b^
	Hexuronic acid (μg/mg protein)	20.8 ± 4.5 ^c^	38.2 ± 5.2 ^a^	30.7 ± 4.6 ^a^	35.9 ± 5.1 ^a^

Values (mean ± SD) were obtained from each group of eight animals. ^a^ *p* < 0.05 and ^b^ *p* < 0.01 compared to the values of rats treated with simple ointment on the indicated post-wounding day, respectively. ^c^ *p* < 0.05 and ^d^ *p* < 0.01 compared to the values of rats treated with nitrofurazone (NF) ointment on the indicated post-wounding day, respectively.

**Table 6:** Effect of test ointments on wound breaking strength (WBS) in incision wound model.

Treatments	WBS (g/mm^2^)	Increases in WBS (%)
Simple ointment	282.3 ± 6.9 ^d^	−
0.2% (*w*/*w*) nitrofurazone ointment	387.6 ± 8.2 ^a^	37.2 ± 6.7
2.5% (*w*/*w*) hispidin ointment	375.2 ± 9.7 ^a^	32.9 ± 5.8
5% (*w*/*w*) hispidin ointment	404.6 ± 10.3 ^b,c^	43.3 ± 5.1 ^c^

Values (mean ± SD) were obtained from each group of eight animals. ^a^ *p* < 0.05 and ^b^ *p* < 0.01 compared to the values of rats treated with simple ointment, respectively. ^c^ *p* < 0.05 and ^d^ *p* < 0.01 compared to the values of rats treated with nitrofurazone ointment, respectively.

## Data Availability

All the data needed to evaluate the conclusions in the paper are present in the paper. Additional data related to this paper may be requested from the authors.

## References

[1] Wilkinson H.N., Hardman M.J. (2020). Wound healing: Cellular mechanisms and pathological outcomes. Open Biol..

[2] Fernández-Guarino M., Hernández-Bule M.L., Bacci S. (2023). Cellular and molecular processes in wound healing. Biomedicines.

[3] Xiao T., Yan Z., Xiao S., Xia Y. (2020). Proinflammatory cytokines regulate epidermal stem cells in wound epithelialization. Stem Cell Res. Ther..

[4] Rousselle P., Braye F., Dayan G. (2019). Re-epithelialization of adult skin wounds: Cellular mechanisms and therapeutic strategies. Adv. Drug Deliv. Rev..

[5] Simões D., Miguel S.P., Ribeiro M.P., Coutinho P., Mendonça A.G., Correia I.J. (2018). Recent advances on antimicrobial wound dressing: A review. Eur. J. Pharm. Biopharm..

[6] Negut I., Grumezescu V., Grumezescu A.M. (2018). Treatment strategies for infected wounds. Molecules.

[7] Bhardwaj S., Mehra P., Dhanjal D.S., Sharma P., Sharma V., Singh R., Nepovimova E., Chopra C., Kuča K. (2022). Antibiotics and antibiotic resistance- flipsides of the same coin. Curr. Pharm. Des..

[8] Trinh X.T., Long N.V., Van Anh L.T., Nga P.T., Giang N.N., Chien P.N., Nam S.Y., Heo C.Y. (2022). A comprehensive review of natural compounds for wound healing: Targeting bioactivity perspective. Int. J. Mol. Sci..

[9] Shevchuk Y., Kuypers K., Janssens G.E. (2023). Fungi as a source of bioactive molecules for the development of longevity medicines. Ageing Res. Rev..

[10] Lu J., Su M., Zhou X., Li D., Niu X., Wang Y. (2024). Research progress of bioactive components in *Sanghuangporus* spp. Molecules.

[11] Palkina K.A., Ipatova D.A., Shakhova E.S., Balakireva A.V., Markina N.M. (2021). Therapeutic potential of hispidin-fungal and plant polyketide. J. Fungi.

[12] Zan L.F., Qin J.C., Zhang Y.M., Yao Y.H., Bao H.Y., Li X. (2011). Antioxidant hispidin derivatives from medicinal mushroom Inonotus hispidus. Chem. Pharm. Bull..

[13] Shao H.J., Jeong J.B., Kim K.J., Lee S.H. (2015). Anti-inflammatory activity of mushroom-derived hispidin through blocking of NF-κB activation. J. Sci. Food Agric..

[14] Lee S., Lee J., Choi K., Kim H., Park Y., Yoon J., Kim J.H., Ryu S. (2021). Polylactic acid and polycaprolactone blended cosmetic microneedle for transdermal hispidin delivery system. Appl. Sci..

[15] Li I.C., Chen C.C., Sheu S.J., Huang I.H., Chen C.C. (2020). Optimized production and safety evaluation of hispidin-enriched *Sanghuangporus sanghuang* mycelia. Food Sci. Nutr..

[16] Balouiri M., Sadiki M., Ibnsouda S.K. (2016). Methods for in vitro evaluating antimicrobial activity: A review. J. Pharm. Anal..

[17] Lai M.C., Liu W.Y., Liou S.S., Liu I.M. (2023). Hispidin in the medicinal fungus protects dopaminergic neurons from JNK activation-regulated mitochondrial-dependent apoptosis in an MPP+-induced in vitro model of Parkinson’s disease. Nutrients.

[18] Singh M., McKenzie K., Ma X. (2017). Effect of dimethyl sulfoxide on in vitro proliferation of skin fibroblast cells. J. Biotech. Res..

[19] Martinotti S., Ranzato E. (2020). Scratch wound healing assay. Methods Mol. Biol..

[20] Murthy S., Gautam M.K., Goel S., Purohit V., Sharma H., Goel R.K. (2013). Evaluation of in vivo wound healing activity of Bacopa monniera on different wound model in rats. Biomed. Res. Int..

[21] Mielke H., Strickland J., Jacobs M., Mehta J. (2017). Biometrical evaluation of the performance of the revised OECD Test Guideline 402 for assessing acute dermal toxicity. Regul. Toxicol. Pharmacol..

[22] Chen W.C., Liou S.S., Tzeng T.F., Lee S.L., Liu I.M. (2012). Wound repair and anti-inflammatory potential of Lonicera japonica in excision wound-induced rats. BMC Complement. Altern. Med..

[23] Fikru A., Makonnen E., Eguale T., Debella A., Mekonnen G.A. (2012). Evaluation of in vivo wound healing activity of methanol extract of *Achyranthes aspera* L.. J. Ethnopharmacol..

[24] Gautam M.K., Purohit V., Agarwal M., Singh A., Goel R.K. (2014). In vivo healing potential of Aegle marmelos in excision, incision, and dead space wound models. Sci. World J..

[25] Altavilla D., Saitta A., Cucinotta D., Galeano M., Deodato B., Colonna M., Torre V., Russo G., Sardella A., Urna G. (2001). Inhibition of lipid peroxidation restores impaired vascular endothelial growth factor expression and stimulates wound healing and angiogenesis in the genetically diabetic mouse. Diabetes.

[26] Tomic-Canic M., Burgess J.L., O’Neill K.E., Strbo N., Pastar I. (2020). Skin microbiota and its interplay with wound healing. Am. J. Clin. Dermatol..

[27] Ding X., Tang Q., Xu Z., Xu Y., Zhang H., Zheng D., Wang S., Tan Q., Maitz J., Maitz P.K. (2022). Challenges and innovations in treating chronic and acute wound infections: From basic science to clinical practice. Burns Trauma.

[28] Ge Y., Wang Q. (2023). Current research on fungi in chronic wounds. Front. Mol. Biosci..

[29] Benedict R.G., Brady L.R. (1972). Antimicrobial activity of mushroom metabolites. J. Pharm. Sci..

[30] Sułkowska-Ziaja K., Trepa M., Olechowska-Jarząb A., Nowak P., Ziaja M., Kała K., Muszyńska B. (2023). Natural compounds of fungal origin with antimicrobial activity-potential cosmetics applications. Pharmaceuticals.

[31] Knoedler S., Broichhausen S., Guo R., Dai R., Knoedler L., Kauke-Navarro M., Diatta F., Pomahac B., Machens H.G., Jiang D. (2023). Fibroblasts—the cellular choreographers of wound healing. Front. Immunol..

[32] Maddaluno L., Urwyler C., Werner S. (2017). Fibroblast growth factors: Key players in regeneration and tissue repair. Development.

[33] Lichtman M.K., Otero-Vinas M., Falanga V. (2016). Transforming growth factor beta (TGF-β) isoforms in wound healing and fibrosis. Wound Repair Regen..

[34] Hardwicke J., Schmaljohann D., Boyce D., Thomas D. (2008). Epidermal growth factor therapy and wound healing—Past, present and future perspectives. Surgeon.

[35] Ahmad A., Nawaz M.I. (2022). Molecular mechanism of VEGF and its role in pathological angiogenesis. J. Cell. Biochem..

[36] Masson-Meyers D.S., Andrade T.A.M., Caetano G.F., Guimaraes F.R., Leite M.N., Leite S.N., Frade M.A.C. (2020). Experimental models and methods for cutaneous wound healing assessment. Int. J. Exp. Pathol..

[37] Gardeazabal L., Izeta A. (2024). Elastin and collagen fibres in cutaneous wound healing. Exp. Dermatol..

[38] Li P., Wu G. (2018). Roles of dietary glycine, proline, and hydroxyproline in collagen synthesis and animal growth. Amino Acids..

[39] Nagaoka I., Igarashi M., Sakamoto K. (2012). Biological activities of glucosamine and its related substances. Adv. Food Nutr. Res..

[40] Landén N.X., Li D., Ståhle M. (2016). Transition from inflammation to proliferation: A critical step during wound healing. Cell Mol. Life Sci..

[41] Holzer-Geissler J.C.J., Schwingenschuh S., Zacharias M., Einsiedler J., Kainz S., Reisenegger P., Holecek C., Hofmann E., Wolff-Winiski B., Fahrngruber H. (2022). The Impact of prolonged inflammation on wound healing. Biomedicines.

[42] Cicchese J.M., Evans S., Hult C., Joslyn L.R., Wessler T., Millar J.A., Marino S., Cilfone N.A., Mattila J.T., Linderman J.J. (2018). Dynamic balance of pro- and anti-inflammatory signals controls disease and limits pathology. Immunol. Rev..

[43] Mahmoud N.N., Hamad K., Al Shibitini A., Juma S., Sharifi S., Gould L., Mahmoudi M. (2024). Investigating inflammatory markers in wound healing: Understanding implications and identifying artifacts. ACS Pharmacol. Transl. Sci..

[44] Short W.D., Rae M., Lu T., Padon B., Prajapati T.J., Faruk F., Olutoye O.O., Yu L., Bollyky P., Keswani S.G. (2023). Endogenous interleukin-10 contributes to wound healing and regulates tissue repair. J. Surg. Res..

[45] Comino-Sanz I.M., López-Franco M.D., Castro B., Pancorbo-Hidalgo P.L. (2021). The role of antioxidants on wound healing: A review of the current evidence. J. Clin. Med..

